# The Anatomy of the Bones, Joints, and Muscles, Exhibiting the Parts as They Appear on Dissection, and More Particularly in the Living Figure; as Applicable to the Fine Arts. Second Edition, in Two Parts

**Published:** 1834-10-01

**Authors:** 


*The Anatomy of the Bones, Joints, and Muscles, exhibiting the Parts as they appear on Dissection, and more particularly in the living Figure; as applicable to the Fine Arts. Second Edition, in two Parts.* By George Simpson, M.R.C.S. &c.— London, 1834. 4to. pp. 141; with 30 Lithographic Drawings.

This work will undoubtedly be useful to artists, though we could have wished that the author had discussed his subject at greater length. It appears to us that he has given the muscles only as they appear on dissection, and *not* as they are seen in the living body. This defect should be supplied when the book is reprinted, and Mr. Simpson should also add references to the works of the great masters, showing how and why particular muscles are brought into play.

The plates of the bones are handsomer than those of the muscles. This arises partly from their having been thrown off on India paper, which gives lithographic plates a relief and a breadth which they are otherwise apt to want; but chiefly from the real superiority of the drawings. These representations of the bones are so uniformly and equably beautiful, that it would be difficult to settle the precedence among them; but perhaps we might point out Plate V., giving a view of the scapula and humerus, and Plate VIII., a front view of the os innominatum and part of the femur, as reflecting the highest credit on the artist, Mr. Haghe. If Mr. Simpson would enrich his next edition with a number of drawings from the life by the same draughtsman, we would assure him a large sale: there is a quantum of merit which is irresistible.

---

*Dispensary Abuses. Addressed to the Profession.*

